# Leveraging Intermolecular Charge Transfer for High-Speed Optical Wireless Communication

**DOI:** 10.1021/acs.jpclett.4c00268

**Published:** 2024-03-08

**Authors:** Xin Zhu, Yue Wang, Issatay Nadinov, Simil Thomas, Luis Gutiérrez-Arzaluz, Tengyue He, Jian-Xin Wang, Omar Alkhazragi, Tien Khee Ng, Osman M. Bakr, Husam N. Alshareef, Boon S. Ooi, Omar F. Mohammed

**Affiliations:** † Advanced Membranes and Porous Materials Center, Division of Physical Science and Engineering, King Abdullah University of Science and Technology, Thuwal 23955-6900, Kingdom of Saudi Arabia; ‡ KAUST Catalysis Center, Division of Physical Sciences and Engineering, King Abdullah University of Science and Technology, Thuwal 23955-6900, Kingdom of Saudi Arabia; § Photonics Laboratory, Division of Computer, Electrical, and Mathematical Sciences and Engineering, King Abdullah University of Science and Technology, Thuwal 23955-6900, Kingdom of Saudi Arabia; ∥ Materials Science and Engineering, Physical Science and Engineering Division, King Abdullah University of Science and Technology (KAUST), Thuwal 23955-6900, Saudi Arabia

## Abstract

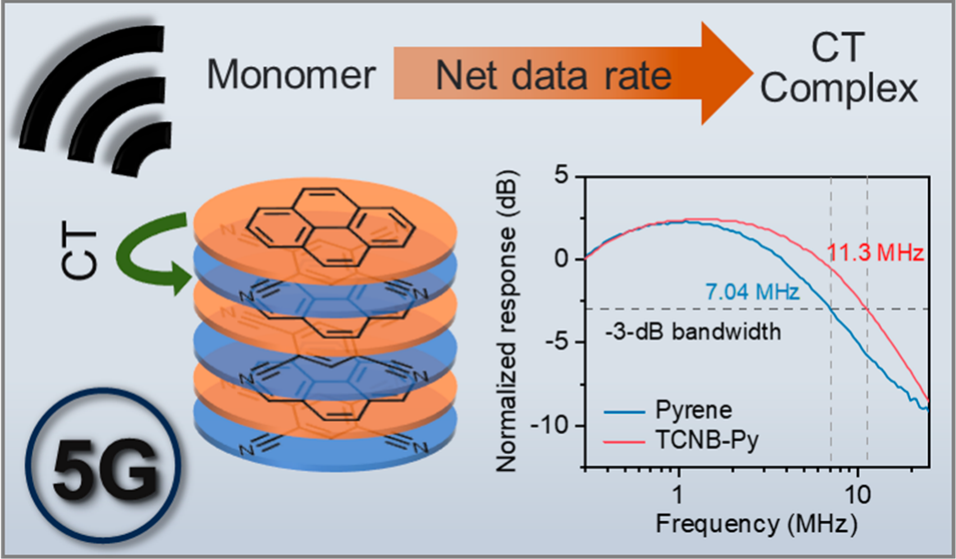

Intermolecular charge transfer (CT) complexes have emerged as versatile platforms with customizable optical properties that play a pivotal role in achieving tunable photoresponsive materials. In this study, we introduce an innovative approach for enhancing the modulation bandwidth and net data rates in optical wireless communications (OWCs) by manipulating combinations of monomeric molecules within intermolecular CT complexes. Concurrently, we extensively investigate the intermolecular charge transfer mechanism through diverse steady-state and ultrafast time-resolved spectral techniques in the mid-infrared range complemented by theoretical calculations using density functional theory. These intermolecular CT complexes empower precise control over the −3 dB bandwidth and net data rates in OWC applications. The resulting color converters exhibit promising performance, achieving a net data rate of ∼100 Mb/s, outperforming conventional materials commonly used in the manufacture of OWC devices. This research underscores the substantial potential of engineering intermolecular charge transfer complexes as an ongoing progression and commercialization within the OWC. This carries profound implications for future initiatives in high-speed and secure data transmission, paving the way for promising endeavors in this area.

As human society’s demand for communication systems and data transmission continuously grows, the prevailing broadband radiofrequency (RF)/microwave wireless technology encounters challenges in keeping pace with the evolving landscape, mainly due to spectrum congestion and limited bandwidth.^1−4^ Nonetheless, the emergence of optical wireless communication (OWC) technology, offering secure, license-free bandwidth across the entire spectrum from ultraviolet (UV) to near-infrared (NIR), presents fresh opportunities for unobstructed growth in the realm of high-speed, low-latency data transmission.^5−11^ Luminescent materials with short lifetimes play an important role in the OWC system, as they can serve as color converters that facilitate white-light generation,^12^ wavelength-division multiplexing/demultiplexing,^13,14^ large-area wide-field-of-view light transmission/collection,^3^ optical beam tracking,^15^ etc., in various forms such as films, fibers, and liquid.^16^ However, conventional color-converting materials utilized for OWCs have primarily revolved around intricate organic, ceramic, and perovskite compositions. The widespread adoption and commercial viability of these materials face substantial obstacles stemming from their convoluted synthesis procedures, increased manufacturing expenses, and considerable toxicity.^17−21^ As a result, the incorporation of innovative materials and strategies into affordable optical communication, aimed at improving modulation bandwidth and increasing data transmission rates, has become an exciting area of research for materials scientists, physicists, and engineers.

The utilization of an intermolecular charge transfer strategy offers a practical and attainable method for surmounting the significant challenges encountered in single molecules and nanomaterial systems.^22−24^ It involves the formation of common organic charge transfer (CT) complexes that exhibit distinct photophysical and morphological functionalities, achieved through diverse noncovalent interactions such as hydrogen bonding and π–π interactions among molecules.^25−27^ Additionally, the materials can be obtained from simple molecular units, obviating the necessity for complex synthetic procedures. These attributes render these complexes promising for applications such as light-emitting diodes, catalysis, and sensing.^28−30^ Furthermore, the intermolecular charge transfer strategy can convert initially nonluminescent substances into luminous materials by generating exciplexes, which further broadens the availability of luminescent materials. Moreover, the incorporation of the charge transfer process typically reduces the luminescence lifetime in comparison to those of monomeric molecules, which proves advantageous for the −3 dB bandwidth in the OWC scenarios. It should be noted that the −3 dB bandwidth (*f*_–3 dB_) and the average luminescence lifetime (⟨τ⟩) are inversely correlated within a certain range, which can be approximately expressed as *f*_–3 dB_ ≤ 1/(2π⟨τ⟩).^13,31,32^ The underlying mechanism relies on the fact that the modulation bandwidth of a luminescent material is limited by its carrier recombination lifetime. A long luminescence lifetime indicates that the carriers take longer to recombine, limiting the speed at which the device can respond to modulation signals. On the contrary, shorter lifetimes allow for faster switching and thus larger modulation bandwidths, which are always pursued for high-speed data transmission.^33^ Consequently, the concept of intermolecular charge transfer introduces a fresh avenue of alternative materials into the realm of OWCs. It holds the potential to furnish novel prospects and impetus for the continued advancement of OWC technology.

In this study, we introduce a series of complexes based on intermolecular charge transfer designed for applications in high-speed OWCs. The intermolecular charge transfer mechanism was comprehensively examined using various steady-state and ultrafast time-resolved spectroscopic techniques supported by density functional theory (DFT) calculations. These CT complexes demonstrate a tunable modulation bandwidth ranging from 7 to 13 MHz, with the net data rate ranging from 40 to 100 Mb/s. This performance demonstrates the viability of employing an intermolecular charge transfer strategy in the context of high-speed OWCs. These findings underscore the immense potential of intermolecular CT complexes in advancing rapid data transmission, offering a highly promising alternative avenue for the evolution of high-performance OWCs, transcending the realm of new material discovery.

1,2,4,5-Tetracyanobenzene (TCNB) and tetrafluoroterephthalonitrile (TFP) were chosen as the electron acceptors due to the potent electron-withdrawing traits of the cyano substituent. Simultaneously, pyrene (Py) was selected as the electron donor, capitalizing on its robust electron-donating ability and extensive π-conjugation structure, which readily facilitates π–π interactions with the electron acceptors (Figure 1a). The CT complexes consisting of pyrene and TCNB (or TFP) were prepared by solution-processed self-assembly (Figure 1b).^34^ Note that upon addition of a mixed solution of TCNB and pyrene to an ethanol/water mixture, cocrystal formation becomes evident within seconds (Figure S1), indicating the successful assembly of monomers into cocrystals. The X-ray diffraction (XRD) pattern of the TCNB-Py and TFP-Py complexes clearly differs from the pattern of each individual constituent molecule (Figure S2), further suggesting the formation of CT complexes. As a result of the exceedingly compact conjugated molecular structures inherent in these two electron acceptors (TCNB and TFP), they do not exhibit absorption and emission spectra under the standard spectroscopic measurement conditions. In contrast, pyrene in the solid form displays a wide-ranging absorption spectrum from 250 to 400 nm (Figure 1c), alongside an emission band from 400 to 550 nm (Figure 1d).

**Figure 1:**
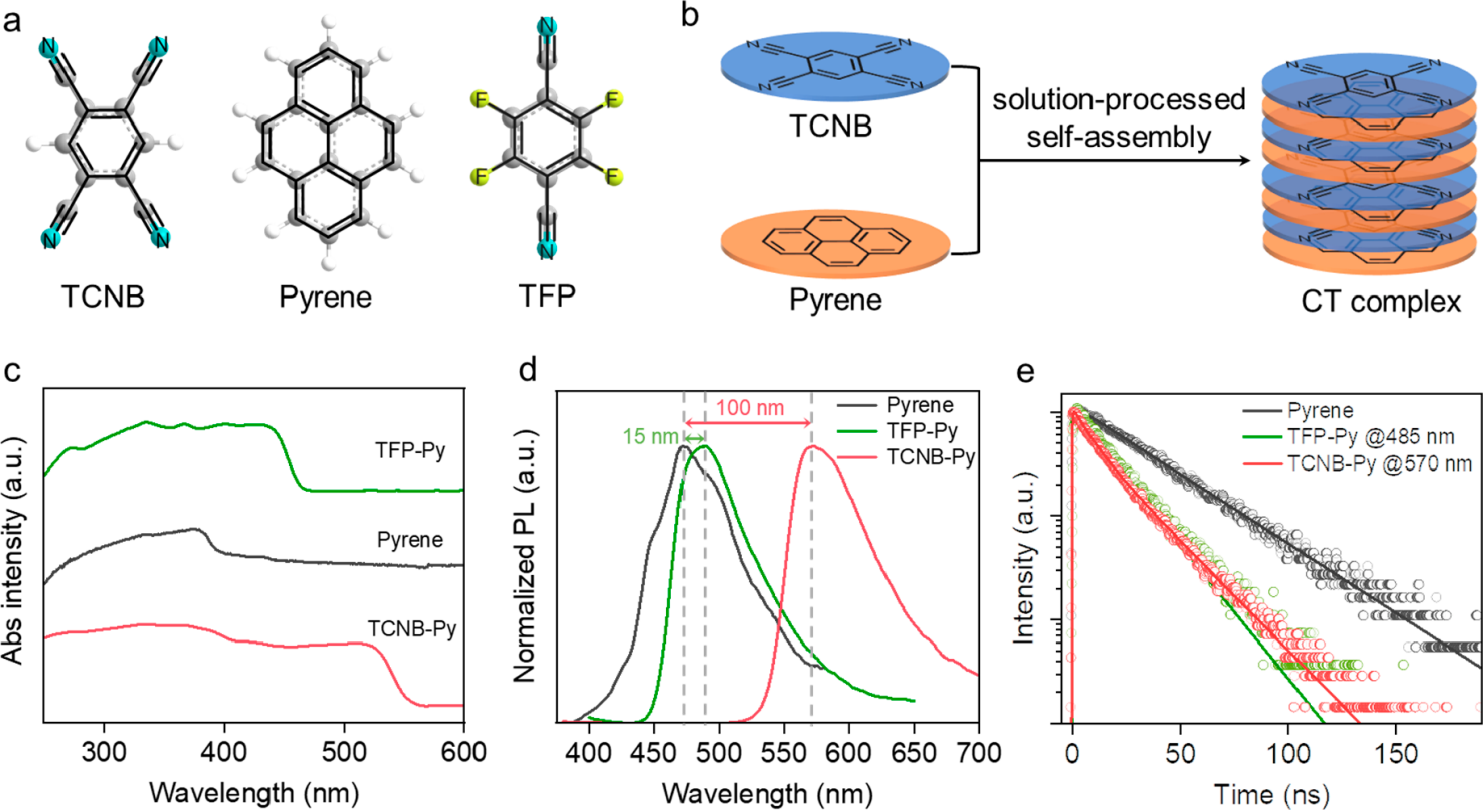
(a) Molecular structures of TCNB, pyrene, and TFP. (b) Illustration of the formation of the intermolecular CT complexes. (c) Absorption, (d) emission (λ_ex_ = 365 nm), and (e) time-correlated single-photon counting (TCSPC) decays of pyrene, TCNB-Py, and TFP-Py.

Remarkably, upon combination of the electron donor and acceptor, the resultant CT complexes (TCNB-Py and TFP-Py) exhibit distinct spectroscopic characteristics. Notably, when compared to those of the individual pyrene components, the complexes show widened absorption spectra, particularly in the longer wavelength range. For instance, the absorption spectrum of TFP-Py extends to 430 nm, and that of TCNB-Py reaches 510 nm, surpassing the excitation range of pyrene on its own. This observation demonstrates the occurrence of an intermolecular charge transfer. Concurrently, the emission spectra of TFP-Py undergo a red-shift from 470 to 485 nm, while the emission spectrum of TCNB-Py experiences a significant red-shift of ∼100 nm, stretching from 470 to 570 nm (Figure 1d). The substantial red-shift in the emission spectra of TCNB-Py can be attributed to the heightened charge transfer propensity induced by the stronger electron-withdrawing ability of the four cyano substituents in the TCNB molecules. Moreover, the photoluminescence lifetime of the CT complexes decreased from 32.5 ± 0.3 ns for the pyrene in isolation to 16.8 ± 0.1 ns (TFP-Py) and 15.1 ± 0.5 ns (TCNB-Py) (Figure 1e). This observation could be attributed to the charge transfer mechanism from pyrene to TFP and TCNB molecules, providing further evidence of the charge transfer process.

To gain insight into the electronic structures of the CT complexes, DFT calculations (Figure 2) were performed. Through analysis of the highest occupied molecular orbital (HOMO) and lowest unoccupied molecular orbital (LUMO) within TCNB-Py and TFP-Py, it becomes evident that the HOMO is localized on the electron donor (Py) whereas the LUMO is localized on the electron acceptor (TCNB/TFP) (Figure 2b,d). The main orbitals contributing to the lowest excitation are from the HOMO → LUMO in both systems, and this lowest excitation involves the electron transfer from the donor unit to the acceptor moiety (Figure 2a,c). Mulliken charge analysis reveals an increase in the negative charge on the TCNB fragment from −0.04 in the ground state (S_0_) to −0.97 in the lowest excited state (S_1_), as well as an increase in the negative charge on the TFP fragment from −0.03 in the S_0_ state to −0.96 in the S_1_ state. Additionally, transition dipole moments (TDMs) of 0.64 D (TCNB-Py) and 1.17 D (TFP-Py) along the molecular stacking direction along with Mulliken charge analysis confirm the charge transfer nature of the lowest excited state. This clearly indicates the presence of a charge transfer mechanism within these complexes.^29^

**Figure 2:**
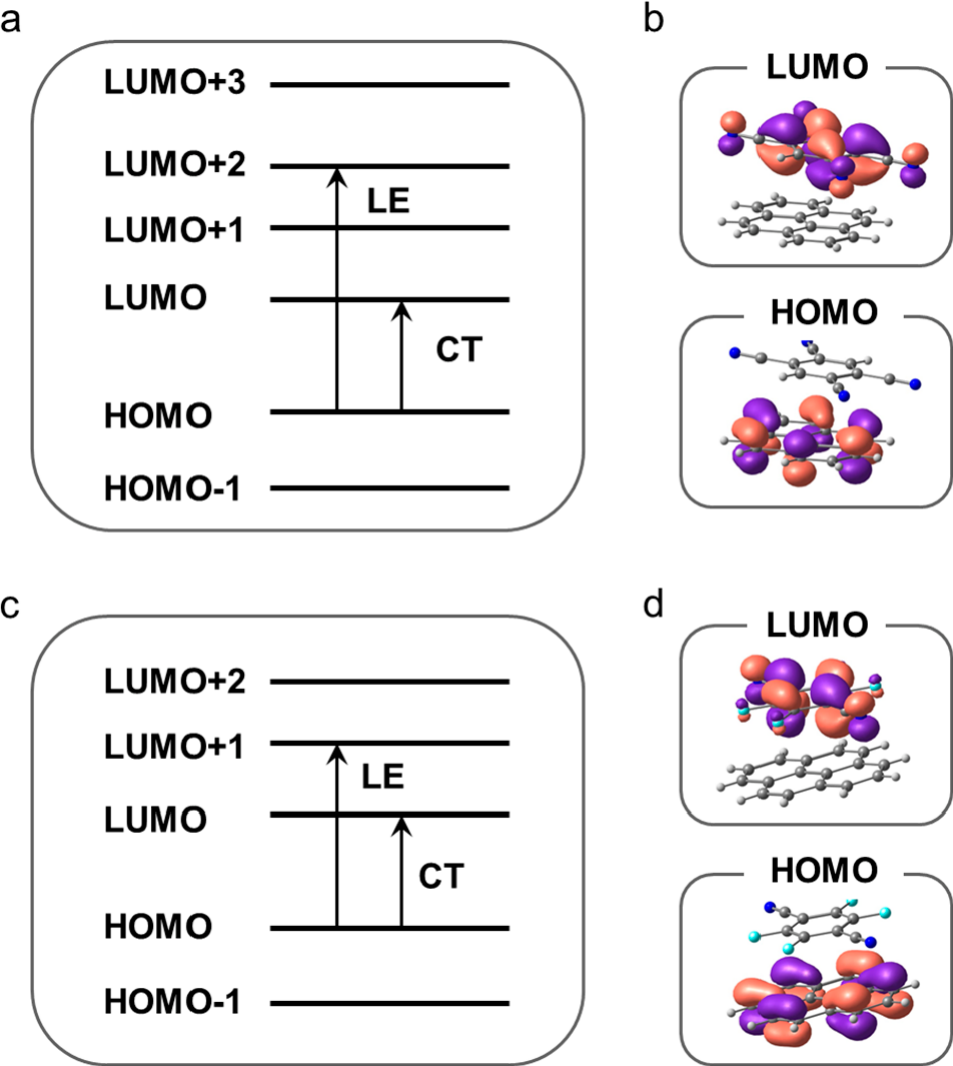
Energy diagrams calculated by the DFT method and corresponding highest occupied molecular orbital (HOMO) and lowest unoccupied molecular orbital (LUMO), with an isovalue of 0.03 au, distributions of (a and b) TCNB-Py and (c and d) TFP-Py (CT is charge transfer excitation, and LE is local excitation).

To investigate the charge transfer mechanism and its dynamics within the TCNB-Py and TFP-Py complexes, we employed time-resolved mid-IR transient absorption spectroscopy. This technique enabled us to track the behavior in real time of the −C≡N stretch vibration of TCNB and TFP in their excited states in real time, providing unique insights into the structural changes occurring within the donor–acceptor complexes during the charge transfer process.^35,36^ Initially, we employed Fourier transform infrared (FT-IR) spectroscopy to discern alterations in the −C≡N vibrational stretch characteristics and quantify the shifts among pure TCNB, TCNB-Py in its ground state, and TCNB-Py in its excited state. A similar approach was employed for TFP and TFP-Py. As depicted in panels a and b of Figure 3, the −C≡N vibrational peaks are located at 2246 and 2243 cm^–1^ for TCNB and TCNB-Py, respectively, while for TFP and TFP-Py, they are located at 2253 and 2248 cm^–1^, respectively. Notably, both TCNB-Py and TFP-Py complexes exhibit red-shifts of 3 and 5 cm^–1^, respectively. This decrease in frequency is indicative of a weakened CN bond within the CT complexes. This weakening of the CN bond highlights alterations in the bonding electronic environment as a result of CT complex formation. The narrowing of the −C≡N vibrational peaks in the presence of pyrene for both TCNB and TFP provides additional evidence of altered molecular conformations resulting from π–π stacking interactions and the formation of CT complexes.

**Figure 3:**
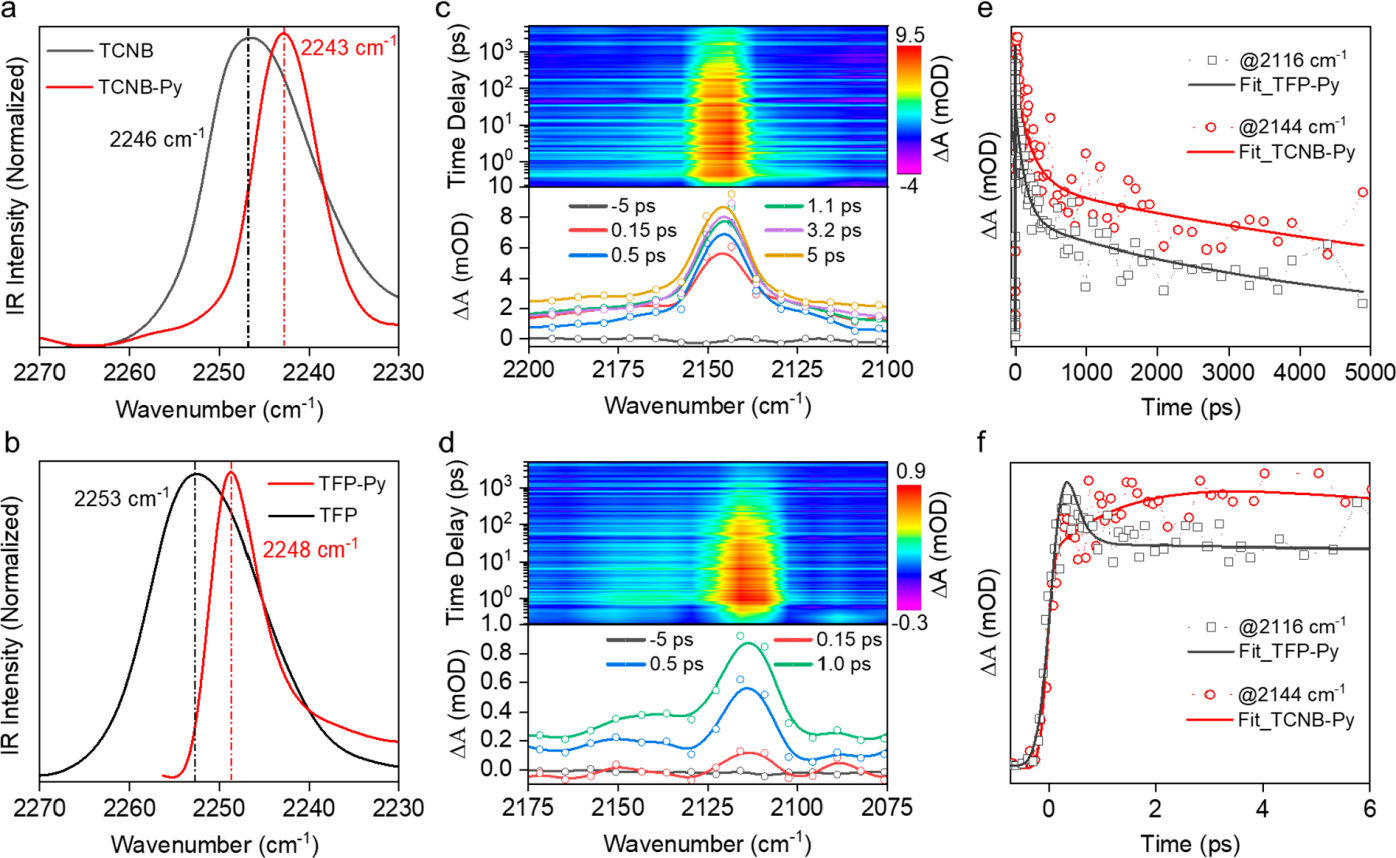
Steady-state FT-IR spectra of (a) TCNB (black) and TCNB-Py (red) and (b) TFP (black) and TFP-Py (red). Map plots of the mid-IR femtosecond spectroscopic measurements and corresponding transient spectra for (c) TCNB-Py and (d) TFP-Py. Kinetic traces of the transient mid-IR −C≡N stretching vibration at 2144 and 2116 cm^–1^ for TCNB-Py (red) and TFP-Py (black) in the (e) long and (f) short time windows.

To study the dynamics of charge transfer, we performed IR transient absorption measurements under 380 nm excitation. The given excitation wavelength has been chosen to selectively excite the donor (Py) and not the acceptors (TCNB and TFP) in TCNB-Py and TFP-Py complexes (Figure S3). IR transient absorption spectra with map plots are given in the range from 2100 to 2200 cm^–1^ for TCNB-Py (Figure 3c) and 2075 to 2175 cm^–1^ for TFP-Py (Figure 3d). Here, we observe new positions for the −C≡N vibration band in the excited state centered at 2144 and 2116 cm^–1^ for TCNB-Py and TFP-Py, respectively, which indicate ∼100 and ∼132 cm^–1^ shifts, respectively, to lower wavenumbers compared to their ground-state positions. Such substantial shifts could be attributed to the redistribution of electron density in the excited state that can lead to changes in bond lengths. This heightened stability likely facilitates a swift and efficient charge transfer process and the anion radical formation of the acceptor, which is evident in CT complexes.^37,38^ Note that this is highlighted by the fact that the pure TCNB and TFP molecules do not exhibit absorption of light at 380 nm and beyond, as illustrated in Figure S3.

Panels e and f of Figure 3 demonstrate a comparison of kinetic traces illustrating the −C≡N vibrational band at 2144 cm^–1^ for TCNB-Py (red curve) and at 2116 cm^–1^ for TFP-Py (black curve). These traces are obtained at various time delays, ranging from −5 ps to 5 ns (Figure 3e) and from −1 to 6 ps (Figure 3f), upon the response to 380 nm excitation. In both kinetic traces, a rising component can be interpreted as a charge transfer process from the pyrene molecule to TCNB or TFP units within the CT complexes. Through curve fitting, time constants of <168 fs [outside of the time resolution of our fs IR-TA system (see Figure S4)] and 1.5 ps (15%) were extracted for TFP-Py and TCNB-Py, respectively. Both time components confirm the ultrafast nature of charge transfer; however, in TFP-Py, this process is even faster. This could be attributed to a more favorable configuration of the donor–acceptor pair, where TFP exhibits a closer energy match with the pyrene molecule. This suitable energy level alignment likely facilitates a faster CT process.

In assessing the applicability of the CT complexes for the purposes of OWC, we performed measurements of the small-signal frequency responses for pyrene, TCNB-Py, and TFP-Py using the setup depicted in Figure 4a. The samples were positioned within an integrating sphere and illuminated by a 375 nm laser diode. The resulting fluorescence emitted from the samples was collected and transformed into an electric signal by an avalanche photodetector (APD). Employing a vector network analyzer (VNA), the modulation signal was introduced to modulate the current of the laser, and the output signal from APD was processed to extract frequency responses across different frequencies. Utilizing sinusoidal alternating current (AC) signals within the frequency spectrum ranging from 300 kHz to 100 MHz, we obtained the −3 dB modulation bandwidths, which stood at 7.04 MHz for pyrene, 11.3 MHz for TCNB-Py, and 12.9 MHz for TFP-Py (as depicted in Figure 4b). These CT complexes exhibited notably broader modulation bandwidths compared to that of the electron donor (Py) that were on par with those of many commercially available ceramic, perovskite, and organic materials.^39−42^ This outcome underscores their considerable potential for applications in high-speed OWCs. Moreover, it is worth highlighting that these CT complexes offer additional advantages over their ceramic and perovskite counterparts, including lower economic and synthesis costs, increased stability and flexibility, and streamlined scalability.

**Figure 4:**
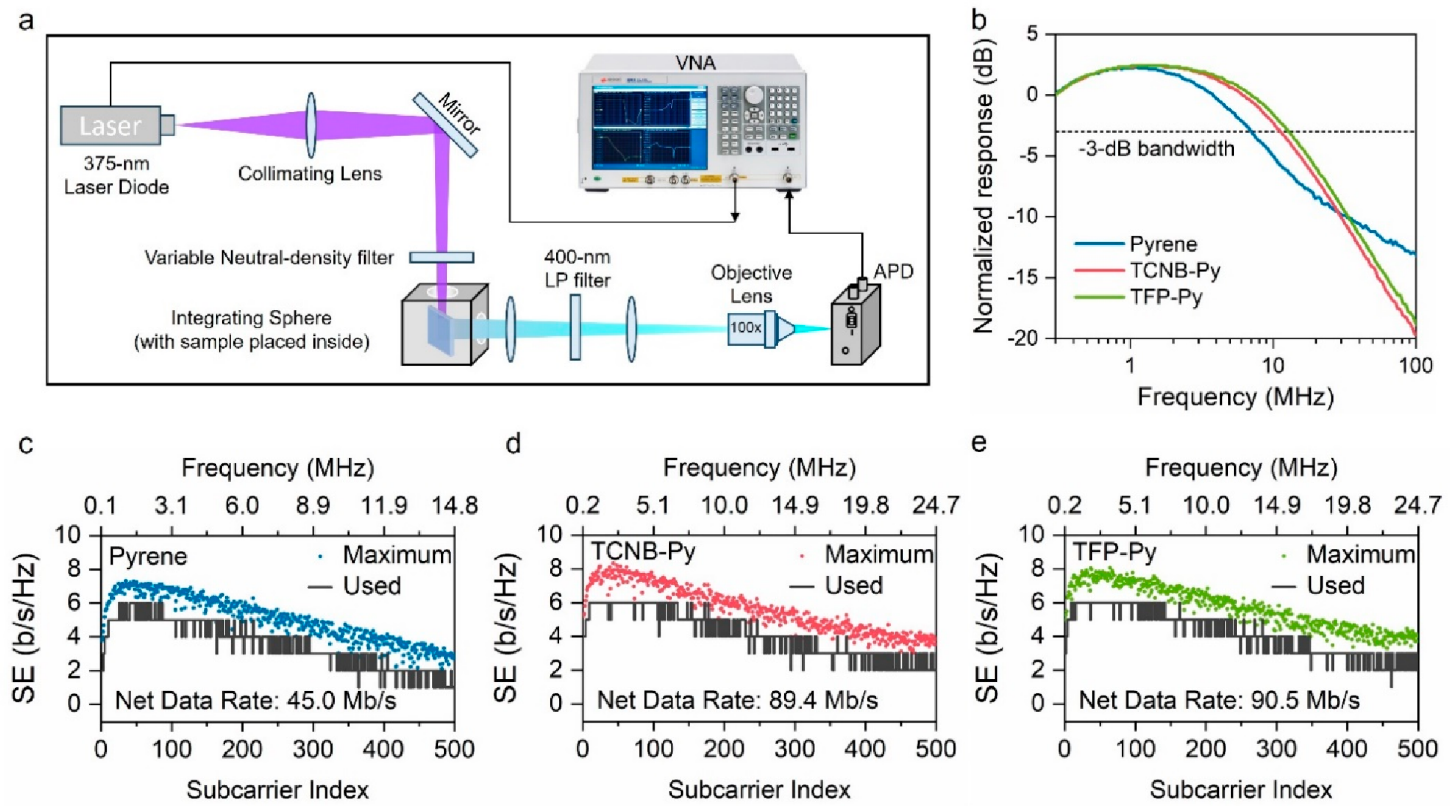
(a) Schematic diagram of the setup for small-signal frequency response measurement and (b) normalized frequency responses of pyrene, TCNB-Py, and TFP-Py, with the −3 dB bandwidths highlighted with a dashed line. Spectral efficiency (SE) of (c) pyrene, (d) TCNB-Py, and (e) TFP-Py during DCO-OFDM implementation.

To further showcase the efficacy of these CT complexes as color converters utilized in OWC links, we conducted direct current-biased optical orthogonal frequency-division multiplexing (DCO-OFDM) modulation. The optical path and the modulation/demodulation steps are shown in Figure S5. As illustrated in Figures S6 and S7, all of the CT complexes exhibited relatively higher signal-to-noise ratios (SNRs) around 25 dB, which allows more signal bits to be loaded beyond their −3 dB bandwidth until 25 MHz. With adaptive power loading and bit allocations, these two CT complexes (TCNB-Py and TFP-Py) exhibited noteworthy net data rates of 89.4 and 90.5 Mb/s, respectively (Figure 4d,e), marking a 2-fold increase compared to that of solely the electron donor (Py) (Figure 4c). These findings provide further validation of the substantial potential of the charge transfer strategy in advancing novel, high-performance color converters tailored to the requirements of OWC applications.

In summary, we have successfully engineered a series of charge transfer complexes designed for high-speed OWC applications. Our investigation of the intermolecular charge transfer mechanism encompassed a comprehensive array of methodologies, including steady-state and ultrafast time-resolved spectroscopic techniques as well as DFT calculations. Notably, these charge transfer complexes with ultrafast transfer rates showcase the capacity for a tunable −3 dB modulation bandwidth and net data rates while demonstrating an OWC performance on par with those of certain conventional materials. This result substantiates the compelling feasibility of adopting an intermolecular charge transfer strategy within the domain of high-speed OWCs. These findings emphasize the significant potential of intermolecular charge transfer complexes in facilitating rapid data transmission. This remarkable discovery presents a highly promising pathway for the advancement of high-performance OWCs, extending beyond the boundaries of novel material exploration.

## Supporting Information Available

Experimental methods and characterization results (PDF)
Transparent Peer Review report available (PDF)

## References

[ref1] Chun H.; Manousiadis P.; Rajbhandari S.; Vithanage D. A.; Faulkner G.; Tsonev D.; McKendry J. J. D.; Videv S.; Enyuan X.; Erdan G.; et al. Visible light communication using a blue GaN μ LED and fluorescent polymer color converter. IEEE Photonics Technol. Lett. 2014, 26, 2035–2038. 10.1109/LPT.2014.2345256.

[ref2] Guo Y.; Kong M.; Sait M.; Marie S.; Alkhazragi O.; Ng T. K.; Ooi B. S. Compact scintillating-fiber/450-nm-laser transceiver for full-duplex underwater wireless optical communication system under turbulence. Opt. Express 2022, 30, 53–69. 10.1364/OE.443800. 35201194

[ref3] Kang C. H.; Alkhazragi O.; Sinatra L.; Alshaibani S.; Wang Y.; Li K.-H.; Kong M.; Lutfullin M.; Bakr O. M.; Ng T. K.; et al. All-inorganic halide-perovskite polymer-fiber-photodetector for high-speed optical wireless communication. Opt. Express 2022, 30, 9823–9840. 10.1364/OE.452370. 35299397

[ref4] Wang J.; Chen C.; Deng B.; Wang Z.; Liu M.; Fu H. Y. Enhancing underwater VLC with spatial division transmission and pairwise coding. Opt. Express 2023, 31, 16812–16832. 10.1364/OE.489530. 37157752

[ref5] Zhu J.; Yu L.; Wang Z.; Wang X.; Ou Y.; Cai M.; Wu Z.; Tang R.; Xia Y.; Wang Y.; et al. High-performance and stable Sb_2_S_3_ thin-film photodetectors for potential application in visible light communication. ACS Appl. Mater. Interfaces 2023, 15, 28175–28183. 10.1021/acsami.3c03671. 37276488

[ref6] Wang J.-X.; Wang Y.; Nadinov I.; Yin J.; Gutiérrez-Arzaluz L.; Alkhazragi O.; He T.; Ng T. K.; Eddaoudi M.; Alshareef H. N.; et al. Aggregation-induced fluorescence enhancement for efficient X-ray imaging scintillators and high-speed optical wireless communication. ACS Mater. Lett. 2022, 4, 1668–1675. 10.1021/acsmaterialslett.2c00498.

[ref7] Shi Y.; Liang D.; Mo Q.; Lu S.; Sun Z.; Xiao H.; Qian Q.; Zang Z. Highly efficient copper-based halide single crystals with violet emission for visible light communication. Chem. Commun. 2023, 59, 583–586. 10.1039/D2CC05965G. 36524689

[ref8] Kang C. H.; Trichili A.; Alkhazragi O.; Zhang H.; Subedi R. C.; Guo Y.; Mitra S.; Shen C.; Roqan I. S.; Ng T. K.; et al. Ultraviolet-to-blue color-converting scintillating-fibers photoreceiver for 375-nm laser-based underwater wireless optical communication. Opt. Express 2019, 27, 30450–30461. 10.1364/OE.27.030450. 31684293

[ref9] Qi Z.; Wang L.; Liu P.; Bai M.; Yu G.; Wang Y. Full-duplex underwater wireless blue light communication. Opt. Express 2023, 31, 9330–9338. 10.1364/OE.483966. 37157505

[ref10] Yu Z.; Huang N.; Li X.; Wang W.; Gong C. Resource-efficient channel estimation for DCO-OFDM based VLC systems. Opt. Express 2023, 31, 27345–27364. 10.1364/OE.494497. 37710813

[ref11] Zhang B.; Chang Y.; Han Z.; Wang W.; Luo B.; Zhai W.; Wang J. Improved dual-polarity response via pyro-phototronic effect for filterless visible light communication. Small 2023, 19, 2207718 10.1002/smll.202207718. 36897011

[ref12] Dursun I.; Shen C.; Parida M. R.; Pan J.; Sarmah S. P.; Priante D.; Alyami N.; Liu J.; Saidaminov M. I.; Alias M. S.; et al. Perovskite nanocrystals as a color converter for visible light communication. ACS Photonics 2016, 3, 1150–1156. 10.1021/acsphotonics.6b00187.

[ref13] Wang J.-X.; Wang Y.; Almalki M.; Yin J.; Shekhah O.; Jia J.; Gutierrez-Arzaluz L.; Cheng Y.; Alkhazragi O.; Maka V. K.; et al. Engineering metal-organic frameworks with tunable colors for high-performance wireless communication. J. Am. Chem. Soc. 2023, 145, 15435–15442. 10.1021/jacs.3c03672. 37421307

[ref14] Manousiadis P. P.; Chun H.; Rajbhandari S.; Vithanage D. A.; Mulyawan R.; Faulkner G.; Haas H.; O’Brien D. C.; Collins S.; Turnbull G. A.; et al. Optical antennas for wavelength division multiplexing in visible light communications beyond the étendue limit. Adv. Opt. Mater. 2020, 8, 1901139 10.1002/adom.201901139.

[ref15] Wang Y.; Wang J.-X.; Alkhazragi O.; Gutiérrez-Arzaluz L.; Zhang H.; Kang C. H.; Ng T. K.; Bakr O. M.; Mohammed O. F.; Ooi B. S. Multifunctional difluoroboron β-diketonate-based luminescent receiver for a high-speed underwater wireless optical communication system. Opt. Express 2023, 31, 32516–32528. 10.1364/OE.500330. 37859053

[ref16] Li X.; Tong Z.; Lyu W.; Chen X.; Yang X.; Zhang Y.; Liu S.; Dai Y.; Zhang Z.; Guo C.; et al. Underwater quasi-omnidirectional wireless optical communication based on perovskite quantum dots. Opt. Express 2022, 30, 1709–1722. 10.1364/OE.448213. 35209331

[ref17] Zhang Y.; Shen W.; Wu S.; Tang W.; Shu Y.; Ma K.; Zhang B.; Zhou P.; Wang S. High-speed transition-metal dichalcogenides based schottky photodiodes for visible and infrared light communication. ACS Nano 2022, 16, 19187–19198. 10.1021/acsnano.2c08394. 36305492

[ref18] Zhu S.; Shan X.; Lin R.; Qiu P.; Wang Z.; Lu X.; Yan L.; Cui X.; Zhang G.; Tian P. Characteristics of GaN-on-Si green micro-LED for wide color gamut display and high-speed visible light communication. ACS Photonics 2023, 10, 92–100. 10.1021/acsphotonics.2c01028.

[ref19] Zhao S.; Jia Z.; Huang Y.; Qian Q.; Lin Q.; Zang Z. Solvent-free synthesis of inorganic rubidium copper halides for efficient wireless light communication and X-ray imaging. Adv. Funct. Mater. 2023, 33, 2305858 10.1002/adfm.202305858.

[ref20] Guo J.; Li Y.; Shan X.; Wang D.; Tian P.; Wang Y. Facile microwave synthesis of efficient green emissive carbon dots powder and their application in visible light communication and white light emitting devices. Adv. Opt. Mater. 2023, 11, 2300984 10.1002/adom.202300984.

[ref21] Sajjad M. T.; Manousiadis P. P.; Orofino C.; Kanibolotsky A. L.; Findlay N. J.; Rajbhandari S.; Vithanage D. A.; Chun H.; Faulkner G. E.; O’Brien D. C.; et al. A saturated red color converter for visible light communication using a blend of star-shaped organic semiconductors. Appl. Phys. Lett. 2017, 110, 013302 10.1063/1.4971823.

[ref22] Zhang D. D.; Suzuki K.; Song X. Z.; Wada Y.; Kubo S.; Duan L.; Kaji H. Thermally activated delayed fluorescent materials combining intra- and intermolecular charge transfers. ACS Appl. Mater. Interfaces 2019, 11, 7192–7198. 10.1021/acsami.8b19428. 30672273

[ref23] Bao L.; Wang B.; Yu P.; Huang C.; Pan C.; Fang H.; Akasaka T.; Guldi D. M.; Lu X. Intermolecular packing and charge transfer in metallofullerene/porphyrin cocrystals. Chem. Commun. 2019, 55, 6018–6021. 10.1039/C9CC02095K. 31062003

[ref24] Yu Y.; Chien S. C.; Sun J.; Hettiaratchy E. C.; Myers R. C.; Lin L. C.; Wu Y. Excimer-mediated intermolecular charge transfer in self-assembled donor-acceptor dyes on metal oxides. J. Am. Chem. Soc. 2019, 141, 8727–8731. 10.1021/jacs.9b03729. 31095391

[ref25] Deng X.; Huang J.; Wang G.; Li J.; Li X.; Lei C.; Zhang K. Enhancing room-temperature phosphorescence via intermolecular charge transfer in dopant-matrix systems. Chem. Commun. 2022, 58, 8137–8140. 10.1039/D2CC03295C. 35775582

[ref26] Montejo M.; Navarro A.; Kearley G. J.; Vázquez J.; López-González J. Intermolecular charge transfer and hydrogen bonding in solid furan. J. Am. Chem. Soc. 2004, 126, 15087–15095. 10.1021/ja040130y. 15548006

[ref27] Sullivan R. P.; Morningstar J. T.; Castellanos-Trejo E.; Bradford R. W.; Hofstetter Y. J.; Vaynzof Y.; Welker M. E.; Jurchescu O. D. Intermolecular charge transfer enhances the performance of molecular rectifiers. Sci. Adv. 2022, 8, eabq7224 10.1126/sciadv.abq7224. 35930649 PMC9355360

[ref28] Sun L.; Zhu W.; Wang W.; Yang F.; Zhang C.; Wang S.; Zhang X.; Li R.; Dong H.; Hu W. Intermolecular charge-transfer interactions facilitate two-photon absorption in styrylpyridine-tetracyanobenzene cocrystals. Angew. Chem., Int. Ed. 2017, 56, 7831–7835. 10.1002/anie.201703439. 28508533

[ref29] Zhu W.; Zheng R.; Fu X.; Fu H.; Shi Q.; Zhen Y.; Dong H.; Hu W. Revealing the charge-transfer interactions in self-assembled organic cocrystals: Two-dimensional photonic applications. Angew. Chem., Int. Ed. 2015, 54, 6785–6789. 10.1002/anie.201501414. 25900165

[ref30] Zhao S.; Xu M.; Liu R.; Xue Y.; Nie J.; Chang Y. NIR-II fluorescent probe for detecting trimethylamine based on intermolecular charge transfer. Chem. - Eur. J. 2022, 28, e202200113 10.1002/chem.202200113. 35324048

[ref31] Kang C. H.; Wang Y.; Alkhazragi O.; Lu H.; Ng T. K.; Ooi B. S. Down-converting luminescent optoelectronics and their applications. APL Photonics 2023, 8, 020903 10.1063/5.0127552.

[ref32] Wang J.-X.; Wang Y.; Nadinov I.; Yin J.; Gutierrez-Arzaluz L.; Healing G.; Alkhazragi O.; Cheng Y.; Jia J.; Alsadun N.; et al. Metal-organic frameworks in mixed-matrix membranes for high-speed visible-light communication. J. Am. Chem. Soc. 2022, 144, 6813–6820. 10.1021/jacs.2c00483. 35412323

[ref33] Wang Y.; Wang H.; Alkhazragi O.; Mohammed Z. O. F.; Gutiérrez-Arzaluz L.; Kang C. H.; Ng T. K.; Ooi B. S. Two-dimensional hybrid organic-inorganic perovskite nanosheets for Gb/s visible-light communication. IEEE Photonics Technol. Lett. 2022, 34, 753–756. 10.1109/LPT.2022.3185843.

[ref34] Lei Y. L.; Jin Y.; Zhou D. Y.; Gu W.; Shi X. B.; Liao L. S.; Lee S. T. White-light emitting microtubes of mixed organic charge-transfer complexes. Adv. Mater. 2012, 24, 5345–5351. 10.1002/adma.201201493. 22833528

[ref35] Gutiérrez-Arzaluz L.; Nadinov I.; Healing G.; Czaban-Jóźwiak J.; Jia J.; Huang Z.; Zhao Y.; Shekhah O.; Schanze K. S.; Eddaoudi M.; et al. Ultrafast aggregation-induced tunable emission enhancement in a benzothiadiazole-based fluorescent metal–organic framework linker. J. Phys. Chem. B 2021, 125, 13298–13308. 10.1021/acs.jpcb.1c08889. 34846146

[ref36] Alomar S. A.; Gutiérrez-Arzaluz L.; Nadinov I.; He R.; Wang X.; Wang J.-X.; Jia J.; Shekhah O.; Eddaoudi M.; Alshareef H. N.; et al. Tunable photoinduced charge transfer at the interface between benzoselenadiazole-based mof linkers and thermally activated delayed fluorescence chromophore. J. Phys. Chem. B 2023, 127, 1819–1827. 10.1021/acs.jpcb.2c08844. 36807993 PMC9986871

[ref37] Mohammed O. F.; Adamczyk K.; Banerji N.; Dreyer J.; Lang B.; Nibbering E. T. J.; Vauthey E. Direct femtosecond observation of tight and loose ion pairs upon photoinduced bimolecular electron transfer. Angew. Chem., Int. Ed. 2008, 47, 9044–9048. 10.1002/anie.200803164. 18932183

[ref38] Mohammed O. F.; Banerji N.; Lang B.; Nibbering E. T. J.; Vauthey E. Photoinduced bimolecular electron transfer investigated by femtosecond time-resolved infrared spectroscopy. J. Phys. Chem. A 2006, 110, 13676–13680. 10.1021/jp066079x. 17181320

[ref39] Mo Q.; Chen C.; Cai W.; Zhao S.; Yan D.; Zang Z. Room temperature synthesis of stable zirconia-coated CsPbBr_3_ nanocrystals for white light-emitting diodes and visible light communication. Laser Photonics Rev. 2021, 15, 2100278 10.1002/lpor.202100278.

[ref40] Lee C.; Shen C.; Oubei H. M.; Cantore M.; Janjua B.; Ng T. K.; Farrell R. M.; El-Desouki M. M.; Speck J. S.; Nakamura S.; et al. 2 Gbit/s data transmission from an unfiltered laser-based phosphor-converted white lighting communication system. Opt. Express 2015, 23, 29779–29787. 10.1364/OE.23.029779. 26698461

[ref41] Wang Z.; Wang Z.; Lin B.; Hu X.; Wei Y.; Zhang C.; An B.; Wang C.; Lin W. Warm-white-light-emitting diode based on a dye-loaded metal-organic framework for fast white-light communication. ACS Appl. Mater. Interfaces 2017, 9, 35253–35259. 10.1021/acsami.7b11277. 28920667

[ref42] Hu X.; Wang Z.; Lin B.; Zhang C.; Cao L.; Wang T.; Zhang J.; Wang C.; Lin W. Two-dimensional metal-organic layers as a bright and processable phosphor for fast white-light communication. Chem. - Eur. J. 2017, 23, 8390–8394. 10.1002/chem.201702037. 28485839

