# Supplementary material for: Leveraging Intermolecular Charge Transfer for High-Speed Optical Wireless Communication
Source: J Phys Chem Lett. 2024 Mar 8;15(11):2988–94. doi: 10.1021/acs.jpclett.4c00268 (PMC10961838; doi:10.1021/acs.jpclett.4c00268)
Supplement: Supplementary file 1 — jz4c00268_si_001.pdf [file jz4c00268_si_001.pdf]

Supporting Information for

Leveraging Intermolecular Charge Transfer for High-Speed Optical Wireless Communication

Xin Zhu,[1] Yue Wang,[3] Issatay Nadinov,[1,4] Simil Thomas,[1] Luis Gutiérrez-Arzaluz,[1,2] Tengyue He,[1] Jian-Xin Wang,[1] Omar Alkhazragi,[3] Tien Khee Ng,[3] Osman M. Bakr,[2] Husam N. Alshareef,[4] Boon S. Ooi,[3] and Omar F. Mohammed[1,2]*

[1]Advanced Membranes and Porous Materials Center, Division of Physical Science and Engineering, King Abdullah University of Science and Technology, Thuwal 23955-6900, Kingdom of Saudi Arabia

[2]KAUST Catalysis Center, Division of Physical Sciences and Engineering, King Abdullah University of Science and Technology, Thuwal 23955-6900, Kingdom of Saudi Arabia

[3]Photonics Laboratory, Division of Computer, Electrical, and Mathematical Sciences and Engineering, King Abdullah University of Science and Technology, Thuwal 23955-6900, Kingdom of Saudi Arabia

[4]Materials Science and Engineering, Physical Science and Engineering Division, King Abdullah University of Science and Technology (KAUST), Thuwal 23955-6900, Saudi Arabia

*Corresponding Authors: Omar F. Mohammed, Email: omar.abdelsaboor@kaust.edu.sa

Materials: Pyrene (97%), tetrafluoroterephthalonitrile (TFP, 98%), and 1,2,4,5-tetracyanobezene (TCNB, 97%) were purchased from Sigma-Aldrich. All chemicals were purchased from commercial suppliers and used without further purification.

Synthesis: The TFP-Py CT complex was synthesized by a liquid-phase self-assembly method. In a typical synthesis, 10 mL of a stock solution of TFP solution in acetonitrile (20 mM) was mixed with an equal volume of pyrene solution in acetonitrile (20 mM) under vigorous stirring. After several minutes, the resultant solution (2.5 mL) was injected into 10 mL of a 9:11 (v/v) ethanol/water mixture. After several minutes, the flocculent suspension appeared within several seconds. The resultant colloidal samples were collected on the surface of a quartz substrate. TCNB-Py complex was synthesized using the same method but instead TCNB with TFP.[1]

Characterization: X-ray diffraction patterns were collected by Bruker D8 ADVANCE diffractometer for Cu Kα radiation (λ = 1.5406 Å) with 2°/ min. UV-vis absorbance studies were carried out with a PerkinElmer Lambda 950 UV/VIS Spectrometer. PerkinElmer LS45 Photoluminescence spectrometer having 450 W Xenon lamp was used for steady-state photoluminescence studies.

Theoretical calculations: The electronic-structure calculations were performed by means of density functional theory (DFT) using the ωB97X-D functional and the 6-31G* basis set. The geometry of the dimers was extracted from the experimental crystal structure. The lowest optical transitions were calculated using time-dependent density functional theory (TD-DFT). The DFT calculations were performed using the Gaussian 16 Revision C.02 suite of programs.[2]

Time-correlated single-photon counting: The time-resolved photoluminescence (PL) experiments for the cocrystals were measured by the Time-Correlated Single-Photon Counting

(TCSPC) technique. TCSPC measurements were performed in a Halcyone setup (Ultrafast Systems). The corresponding excitation wavelengths were selected using a parametric optical amplifier (Newport, Spectra-Physics) that was pumped with an Astrella femtosecond pulsed laser (800 nm, 150 fs, 1 kHz, Coherent). Photoluminescence at different wavelengths was collected and recollimated by a pair of parabolic mirrors passed through a long-pass filter (422 nm, Newport) and finally focused on an optical fiber coupled to a monochromator and a PMT detector. The energy at each excitation wavelength was set constant with the help of a pair of variable neutral density filters (Thorlabs) to ensure that less than 1% of excitation events resulted in a detected photon. TCSPC histograms were fitted using the Lavenberg-Marquart algorithm implemented in Ultrafast System software. The overall time resolution for the system was better than 150 ps.

Time-resolved mid-IR spectroscopy: Time-resolved mid-IR experiments were performed using a Helios-IR spectrometer with broadband capabilities (Ultrafast Systems). The pump pulses at 380 nm were generated through the second harmonic generation of a 120 fs Ti:sapphire regenerative amplifier operating at 1 kHz and emitting at 800 nm (Astrella laser system, Coherent Inc). The tunable mid-IR probe pulses at 4550 nm (2200 cm^{-1}) were produced via difference-frequency mixing in a near-IR optical parametric amplifier (Topas prime–Light Conversion/Spectra-Physics). Comprehensive details of the experimental setup can be found elsewhere.[3] During the transient IR measurements, the photoinduced processes were recorded for TCNB-Py and TFP-Py co-crystal samples placed between two CaF_2 cell windows. To eliminate ambient light, the spectrometer setup was appropriately shielded.

Small-signal frequency response: A 375-nm laser diode (LD) (Nichia, NDU4116) was used as the transmitter. The diode was installed in a laser diode mount (Thorlabs, LDM56F/M), including an integrated thermo-electric cooler, temperature controller, and bias-tee. The temperature of the

LD operation is maintained at 21 °C. The RF signal at different frequencies was generated by a vector network analyzer (VNA) (Agilent Technologies, E5061B) to modulate the driving current of LD. The modulated laser beam was collimated and guided into the integrating sphere to excite the samples. Then, the scattered output light was focused by a series of aspheric condensers and an objective lens. A 400-nm long-pass (LP) filter (Thorlabs, FELH0400) was mounted between the two aspheric condensers to ensure that no unabsorbed photons emitted by the excitation source are detected by the photodetector. A silicon-based avalanche photodetector (Thorlabs, APD430A2/M) with an active diameter of 0.2 mm and an output –3-dB bandwidth of up to 400 MHz was mounted after the objective lens as a receiver. The electrical signal from the APD was analyzed by the VNA to obtain the frequency response information.

DC-biased optical orthogonal frequency-division multiplexing (DCO-OFDM): When carrying out the experiment, the input RF signal was provided by an arbitrary waveform generator (AWG) (Siglent, SDG6052X), and the output signal was recorded by a mixed-domain oscilloscope (Tektronix, MDO3000). The recorded signed signal was then exported and processed offline by MATLAB.

As shown in Figure S5, 2^{16}-1 pseudorandom binary sequence (PRBS) is generated and converted into an array whose size is defined by the number of used subcarriers (500) and the number of OFDM symbols to be transmitted (150). After performing quadrature amplitude modulation (QAM), Hermitian symmetry is imposed to ensure that the output of the inverse fast Fourier transform (IFFT) in the following step is real-valued, which is necessary for intensity modulation/direct detection (IM/DD) systems. A cyclic prefix of length 10 is added to minimize inter-symbol interference (ISI) and approximate circular convolution with the impulse response of the channel, resulting in multiplication in the frequency domain, which simplifies applying single-

tap equalization based on channel estimation from the training symbols. The parallel sequence is then converted back into a serial sequence before transmission through the AWG, whose sampling rates (f_{AWG}) were set to 30 MSamples/s for Pyrene, 50 MSamples/s for TCNB-Py and TFP-Py.

The oscilloscope sampling rate is set to 50 MSamples/s for Pyrene, 125 MSamples/s for TCNB-Py and TFP-Py. The received signals were then synchronized after correlation with training symbols. After synchronization, the signal is converted to a parallel sequence and FFT is performed after removing the cyclic prefix. The symbols added for the Hermitian symmetry are then removed and single-tap equalization is applied to the signal. The QAM symbols (Figure S7) are then demodulated, and the output is converted to a serial sequence for comparison with the transmitted signal to calculate the bit error ratio (BER), which is shown for each subcarrier in Figure S6.

Results and Discussion

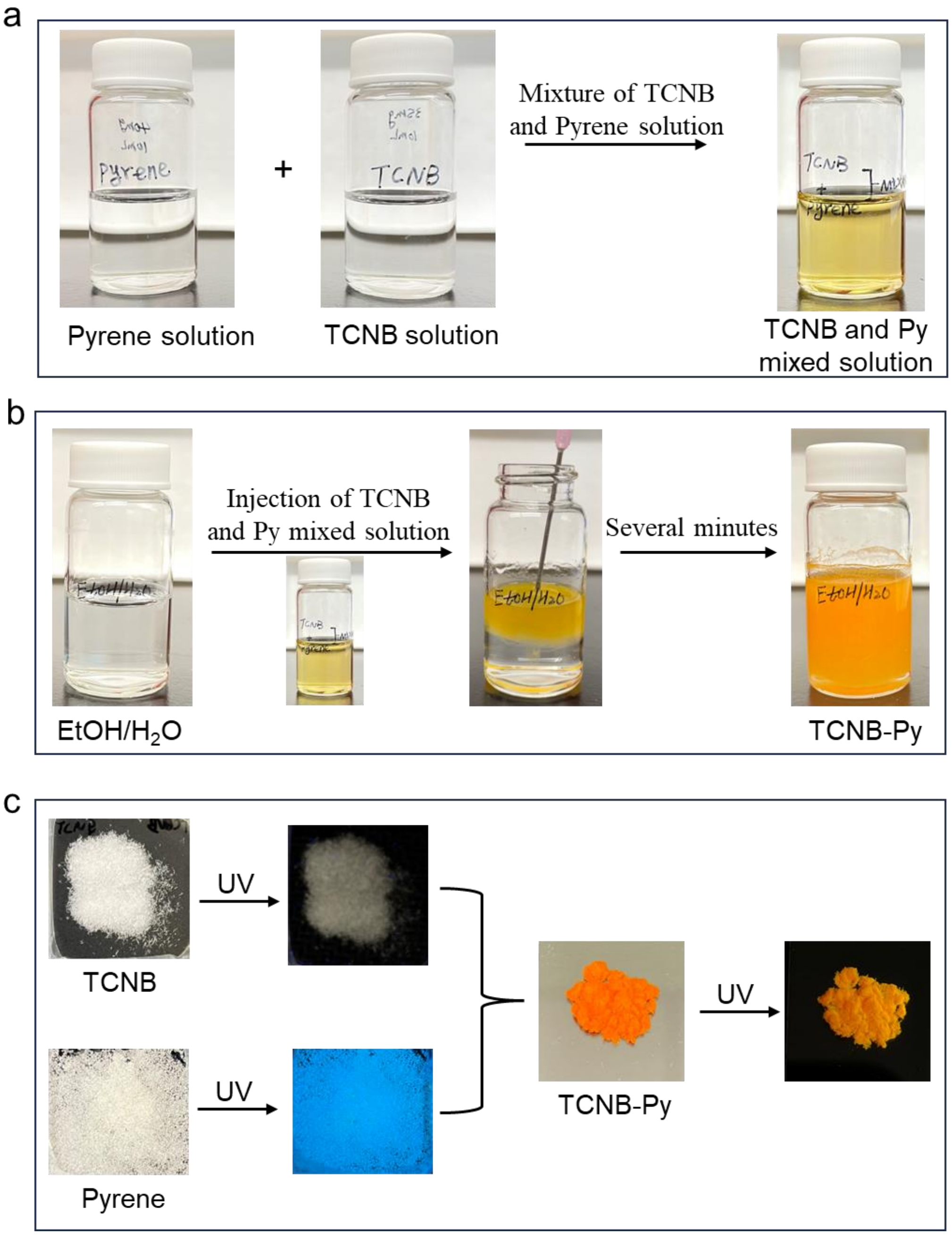

Figure S1. (a) Solutions of TCNB, Pyrene, and TCNB and Pyrene in acetonitrile. (b) The formation process of TCNB-Py cocrystals in ethanol/water mixed solution. (c) The photographs of TCNB, Pyrene, and TCNB-Py cocrystals under sunlight and UV excitation.

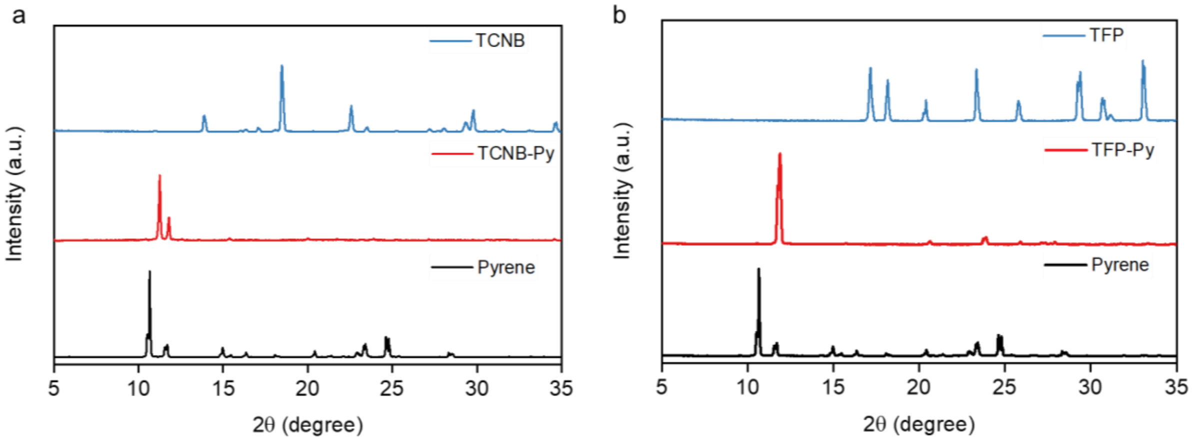

Figure S2. XRD patterns of (a) TCNB, Pyrene and TCNB-Py cocrystals, and (b) TFP, Pyrene and TFP-Py cocrystals.

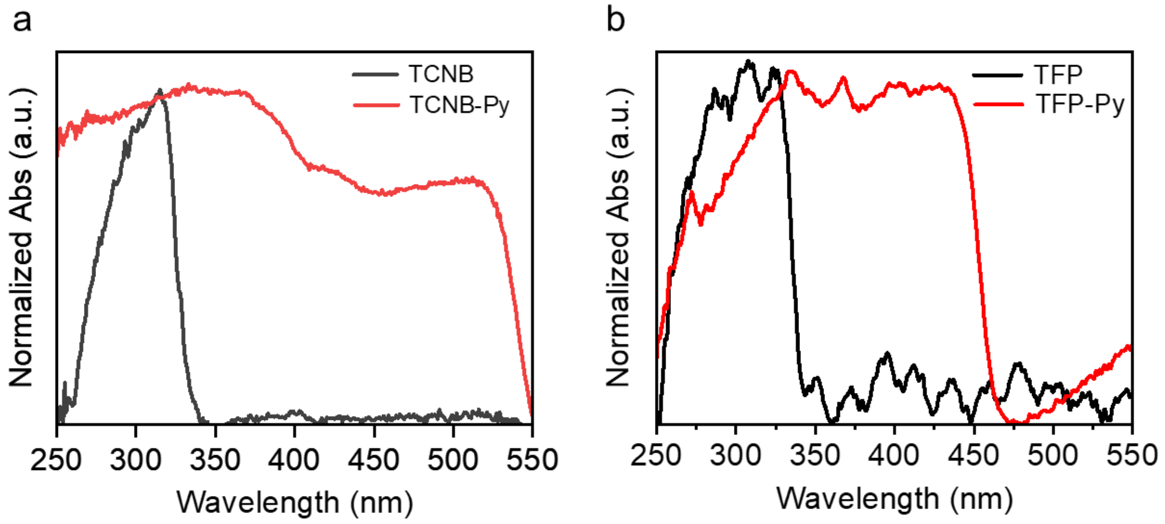

Figure S3. The absorption spectra of (a) TCNB-Py, (b) TFP-Py complex, and their respective constituent materials (TCNB, TFP).

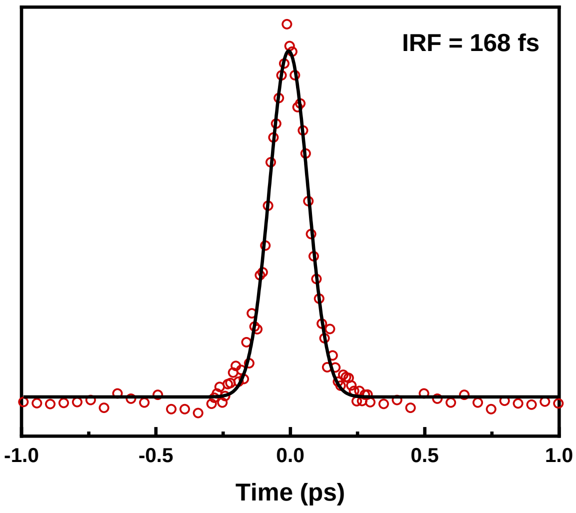

Figure S4. The instrument response function (IRF) for the TA measurements is 168 fs, determined through methanol scattering measurements.

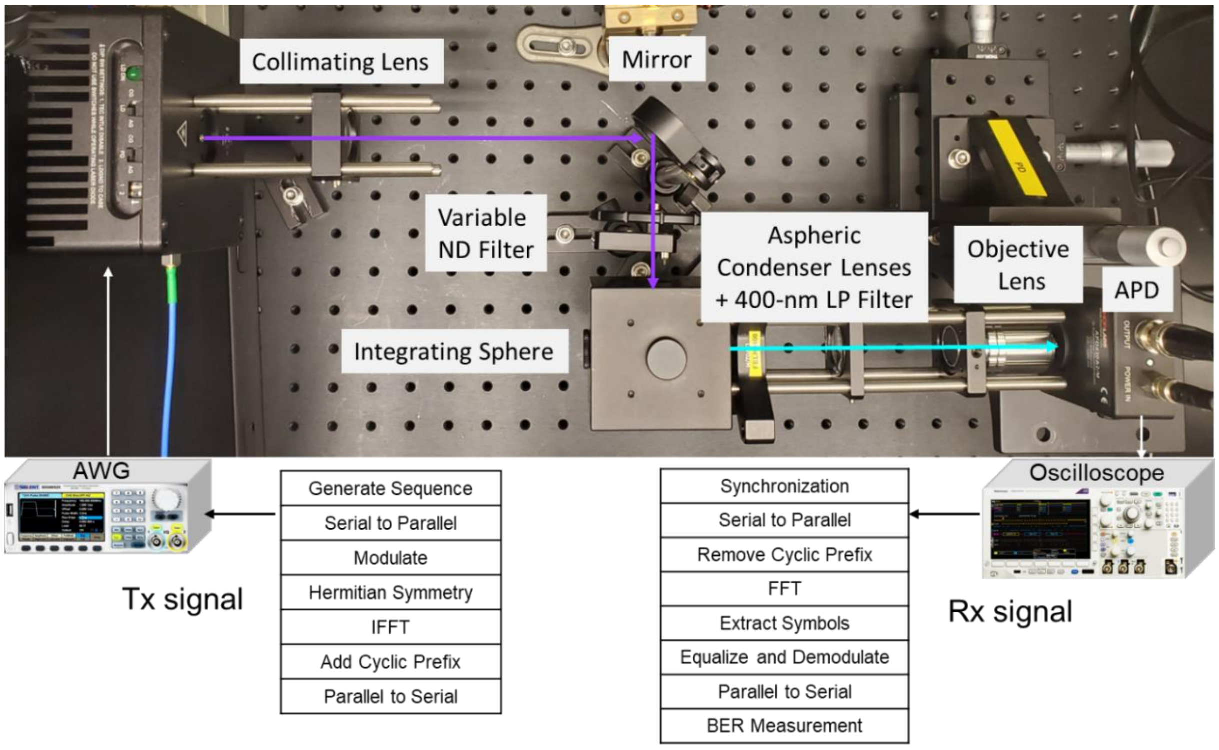

Figure S5. Photograph of the optical path established for small-signal frequency response and DCO-OFDM measurement with the block diagram of modulation and demodulation steps.

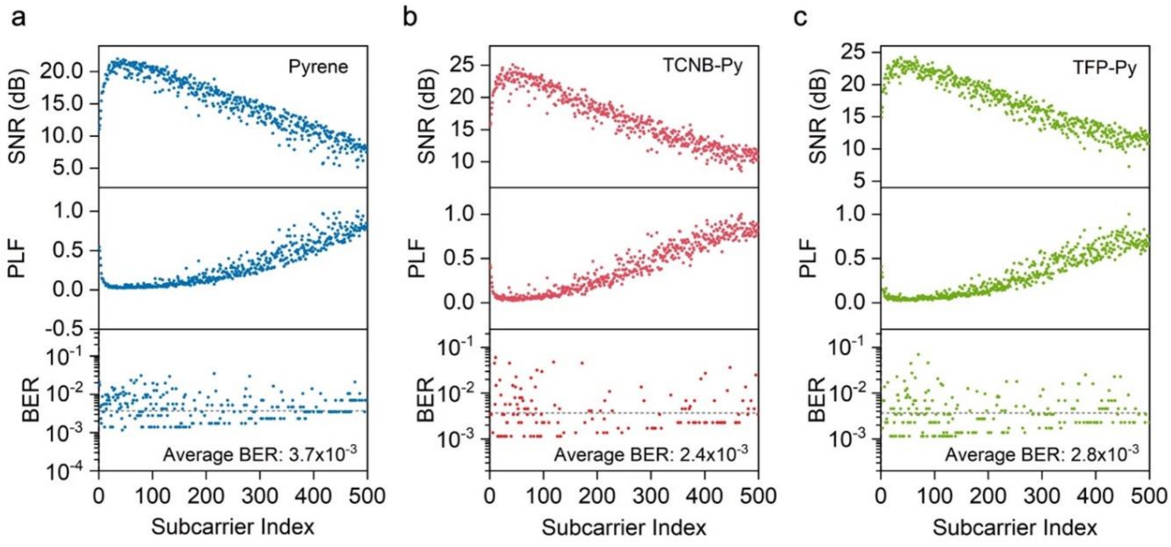

Figure S6. The pertinent metrics for each subcarrier, including estimated signal-to-noise ratio (SNR), power loading factor (PLF), and bit error ratio (BER) of (a) Pyrene, (b) TCNB-Py, and (c) TFP-Py.

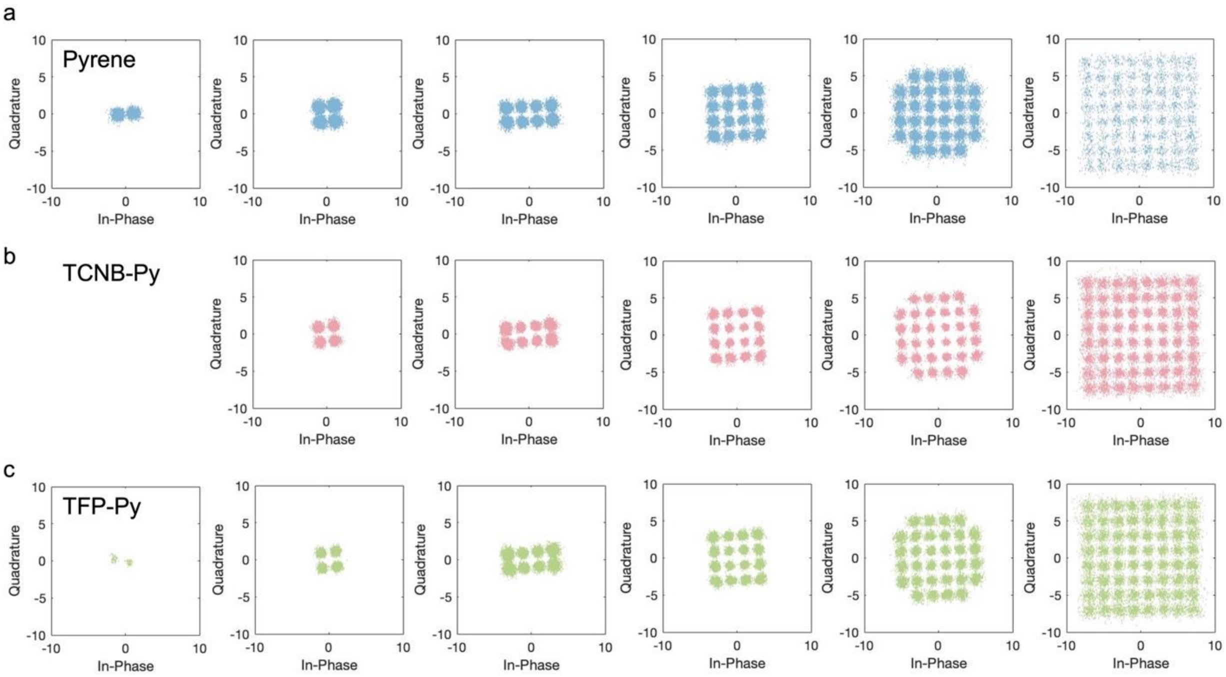

Figure S7. The constellation diagrams from 2 QAM to 64 QAM of (a) Pyrene, (b) TCNB-Py, and (c) TFP-Py.

Reference

(1) Sun, Y.; Lei, Y.; Liao, L.; Hu, W. Competition between arene-perfluoroarene and charge-transfer interactions in organic light-harvesting systems. *Angew. Chem. Int. Ed.* **2017**, *56*, 10352–10356.

(2) Frisch, M. J.; Trucks, G. W.; Schlegel, H. B.; Scuseria, G. E.; Robb, M. A.; Cheeseman, J. R.; Scalmani, G.; Barone, V.; Petersson, G. A.; Nakatsuji, H.; Li, X.; Caricato, M.; Marenich, A. V.; Bloino, J.; Janesko, B. G.; Gomperts, R.; Mennucci, B.; Hratchian, H. P.; Ortiz, J. V.; Izmaylov, A. F.; Sonnenberg, J. L.; Williams-Young, D.; Ding, F.; Lipparini, F.; Egidi, F.; Goings, J.; Peng, B.; Petrone, A.; Henderson, T.; Ranasinghe, D.; Zakrzewski, V. G.; Gao, J.; Rega, N.; Zheng, G.; Liang, W.; Hada, M.; Ehara, M.; Toyota, K.; Fukuda, R.; Hasegawa, J.; Ishida, M.; Nakajima, T.; Honda, Y.; Kitao, O.; Nakai, H.; Vreven, T.; Throssell, K.; Montgomery, J. A., Jr.; Peralta, J. E.; Ogliaro, F.; Bearpark, M. J.; Heyd, J. J.; Brothers, E. N.; Kudin, K. N.; Staroverov, V. N.; Keith, T. A.; Kobayashi, R.; Normand, J.; Raghavachari, K.; Rendell, A. P.; Burant, J. C.; Iyengar, S. S.; Tomasi, J.; Cossi, M.; Millam, J. M.; Klene, M.; Adamo, C.; Cammi, R.; Ochterski, J. W.; Martin, R. L.; Morokuma, K.; Farkas, O.; Foresman, J. B.; Fox, D. J. Gaussian 16, Revision C.02, Gaussian, Inc., Wallingford CT, 2016.

(3) Alsam, A. A.; Aly, S. M.; Usman, A.; Parida, M. R.; Del Gobbo, S.; Alarousu, E.; Mohammed, O. F. Bimolecular excited-state electron transfer with surprisingly long-lived radical ions. *J. Phys. Chem. C* **2015**, *119*, 21896–21903.
