# Supplementary material for: Leveraging Intermolecular Charge Transfer for High-Speed Optical Wireless Communication
Source: J Phys Chem Lett. 2024 Mar 8;15(11):2988–94. doi: 10.1021/acs.jpclett.4c00268 (PMC10961838; doi:10.1021/acs.jpclett.4c00268)
Supplement: Supplementary file 2 — jz4c00268_si_002.pdf [file jz4c00268_si_002.pdf]

jz-2024-00268z.R1

Name: Peer Review Information for "Leveraging Intermolecular Charge Transfer for High-Speed Optical Wireless Communication"

First Round of Reviewer Comments

Reviewer: 1

Comments to the Author
In this paper, Zhu et al. employ intermolecular charge transfer (CT) strategies to demonstrate enhancement of bandwidth modulation and net data rates in optical wireless communication (OWC) applications. While the overall premise of their studies appears reasonable, their submission in its present form, needs improvement to justify publication, i.e., a major revision is necessary. The authors should address the following concerns:

1. The authors write on page3, ‘the incorporation of the charge transfer process typically reduces the luminescence lifetime in comparison to monomeric molecules, which proves advantageous for the –3-dB bandwidth in OWC scenarios.’ The authors should briefly explain the above, as the general reader of JPCL may not be familiar with the technical aspects of OWC and fail to grasp the implication of such a statement.

2. The emission of TCNB-Py exhibits a larger redshift than in TFP-Py. The authors attribute this to the stronger electron-withdrawing ability of 4 cyano groups. In contrast, why is the Py lifetime quenched to a greater extent in TFP-Py, compared to TCNB-Py?

3. In Figs 3 a) and b), why do the IR peaks show narrower spectral width in presence of Py?

4. What are the amplitudes associated with the ultrafast time constants (<150 fs and 1.5 ps) extracted for TFP-Py and TCNB-Py. What is the IRF of the IR transient absorption setup?

5. In light of the fact that ground state redshifts of 3-5 wavenumbers have been attributed to the weakening of the CN bond within CT complexes, are the huge shifts by 100-132 wavenumbers following photoexcitation of Py, also indicative of the same phenomena (CT)? Are these values typical for the nitrile stretch?

Reviewer: 2

Comments to the Author
This letter presents a series of charge transfer complexes to implement for high-speed optical wireless communication applications. The intermolecular charge transfer mechanism is proposed here with support of steady-state and ultrafast time-resolved spectroscopy, and also DFT calculations. The overall manuscript is informative with new physical insights, and may be considered for JPCL. I have couple of questions and hope, these would help authors to improve the manuscript.

1. Figure 3 FTIR data: Are these make for same intensity or have same intensity?
2. The time constant variations in TFP-Py and TCNB-Py may be explained.
3. Can the solution assembly be microscopically viewed? Or any strong other evidences be provided?

Author's Response to Peer Review Comments:

Reviewer: 1

In this paper, Zhu et al. employ intermolecular charge transfer (CT) strategies to demonstrate enhancement of bandwidth modulation and net data rates in optical wireless communication (OWC) applications. While the overall premise of their studies appears reasonable, their submission in its present form, needs improvement to justify publication, i.e., a major revision is necessary. The authors should address the following concerns:

Response: We are grateful for the reviewer's thoughtful insights and appreciate the valuable suggestions aimed at enhancing the quality of our manuscript. Specifically, Reviewer 1 offered invaluable feedback on the charge transfer mechanism and OWC applications sections to improve the clarity of our work. We have fully reviewed and implemented each of these suggestions in the revised version of the manuscript. Below, we provide a detailed response to the reviewer's comments, addressing them point-by-point.

Q1. The authors write on page3, 'the incorporation of the charge transfer process typically reduces the luminescence lifetime in comparison to monomeric molecules, which proves advantageous for the –3-dB bandwidth in OWC scenarios.' The authors should briefly explain the above, as the general reader of JPCL may not be familiar with the technical aspects of OWC and fail to grasp the implication of such a statement.

Response: We thank the reviewer for this remark. More explanation and reference have been added on Page 3 to explain the relationship between luminescence lifetime and modulation bandwidth as follows: "Normally, the –3-dB bandwidth ($f_{-3\text{-dB}}$) and the average luminescence lifetime ($<\tau>$) are inversely correlated within a certain range, which can be approximately expressed as $f_{-3\text{-dB}} \leq 1/(2\pi<\tau>)$. The underlying mechanism relies on the fact that the modulation bandwidth of a luminescent material is limited by its carrier recombination. A long luminescence lifetime indicates that the carriers take longer to recombine, limiting the speed at which the device can respond to modulation signals. On the contrary, shorter lifetimes allow for faster switching and thus higher modulation bandwidths, which are always pursued for high-speed data transmission. (*IEEE Photonics Technol. Lett.* **2022**, *34*, 753–756)". We have fully clarify this issue in the revised version of the manuscript (see page 3).

Q2. The emission of TCNB-Py exhibits a larger redshift than in TFP-Py. The authors attribute this to the stronger electron-withdrawing ability of 4 cyano groups. In contrast, why is the Py lifetime quenched to a greater extent in TFP-Py, compared to TCNB-Py?

Response: We thank the reviewer for this remark. We're sorry for such wrong statement concerning the lifetime of TCNB-Py. Upon re-evaluation, the lifetime of TCNB-Py is determined to be 15.1 ± 0.5 ns (τ_1=7.53 ± 0.5 ns (41.6%), τ_2= 20.6 ± 0.6 ns (58.4%)) using a bi-exponential function, which is slightly shorter compared to TFP-Py (16.8 ± 0.1 ns). This suggests that the stronger charge transfer interaction in TCNB-Py significantly quenches the lifetime of Py, indicating a more pronounced impact on the luminescent lifetime compared to TFP-Py. This result is consistent with the observed emission shift. We have fixed this issue in the revised version of the manuscript (See page 6).

Q3. In Figs 3 a) and b), why do the IR peaks show narrower spectral width in presence of Py?

Response: We thank the reviewer for pointing this out. We would like to clarify that the Py is known for its ability to undergo π-π stacking interactions with other aromatic molecules. This interaction may lead to a stabilization of certain molecular conformations or a reduction in the vibrational energy levels associated with specific bonds with specific charge localization around

the C triple bond N in our system, resulting in narrowing of the IR peaks. To further elucidate this issue, we have expanded the discussion in the revised version of the manuscript (See page 8).

Q4. What are the amplitudes associated with the ultrafast time constants (<150 fs and 1.5 ps) extracted for TFP-Py and TCNB-Py. What is the IRF of the IR transient absorption setup?
Response: We appreciate the reviewer for bringing this to our attention. The instrument response function (IRF) for the TA measurements is 168 fs, determined through methanol scattering measurements, as depicted in the figure below. For TCNB-Py, the amplitude associated with the time constant of 1.5 ps is 15%. Regarding TFP-Py, its time constant falls out of the resolution of our IR system, specifically <168 fs. Consequently, we have updated the extracted time constant for TFP-Py from "<150 fs" to "<168 fs", and added the amplitude value for the extracted time constant for TCNB-Py (See page 9). Furthermore, we acknowledge the reviewer's suggestion regarding the importance of showcasing the IRF within the paper and have made provisions to include the IRF data of the TA measurements in the revised version of the SI (Figure S4).

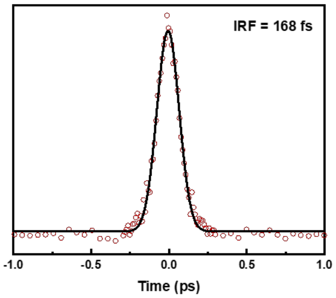

Q5. In light of the fact that ground state redshifts of 3-5 wavenumbers have been attributed to the weakening of the CN bond within CT complexes, are the huge shifts by 100-132 wavenumbers following photoexcitation of Py, also indicative of the same phenomena (CT)? Are these values typical for the nitrile stretch?
Response: We appreciate the reviewer's comment. The significant shift, such as the observed shifts of 100 or 132 cm^{-1} shift, can be attributed to several factors. One of the key factors is charge transfer (CT), which can alter the distribution of electron density within the molecular complex. This redistribution of electron density can lead to changes in bond lengths and strengths, subsequently resulting in shifts in the CN vibrational frequency. Another contributing factor may involve changes in molecular geometry, including alterations in bond angles or distances. Such geometric changes can influence the force constants of vibrational modes, further contributing to observed shifts in CN bands. However, according to the DFT calculation, we observe a large charge transfer from Py to TCNB, and from Py to TFP. Therefore, we may conclude that the observed shift is mainly attributed to the CT phenomenon in our system. To fully address this comment, we expanded the explanation in the revised version of the manuscript (See page 8).

Reviewer: 2
This letter presents a series of charge transfer complexes to implement for high-speed optical wireless communication applications. The intermolecular charge transfer mechanism is proposed here with support of steady-state and ultrafast time-resolved spectroscopy, and also DFT calculations. The overall manuscript is informative with new physical insights, and may be

considered for JPCL. I have couple of questions and hope, these would help authors to improve the manuscript.

Response: We thank the reviewer for appreciating the importance of our work and for recommending publication in JPCL after minor revision. Reviewer 2 raised some minor concerns for clarification to improve the presentation of our manuscript, and we have fully addressed these concerns in the revised version of the manuscript. Here is our response to the comments of the reviewer in a point-by-point fashion.

Q1. Figure 3 FTIR data: Are these make for same intensity or have same intensity?

Response: We appreciate the reviewer's comment. It is noted that the intensities for the CN band presented in Figures 3a and 3b have been normalized to enhance clarity regarding the observed shifts in wavenumbers and narrowing of the band. In response to this feedback, we have revised the Y-axis label to "IR Intensity (Normalized)" in both figures, aiming to provide further elucidation on this matter in the revised version of the manuscript (See page 7).

Q2. The time constant variations in TFP-Py and TCNB-Py may be explained.

Response: We appreciate the reviewer's input. In our experimental approach, we utilized the fitted kinetic traces of the CN vibrational band for TFP-Py and TCNB-Py. From these traces, we extracted time constants for the rising components. For TFP-Py, the extracted time constant is <168 fs, which falls beyond the time resolution of our femtosecond infrared transient absorption. For TCNB-Py, the extracted time constant is 1.5 ps, representing 15% of the total intensity. We attribute these rising components to the charge transfer from Py to TCNB and from Py to TFP units within CT complexes. Both of these time components demonstrate the ultrafast nature of the CT. Interestingly, in the case of TFP-Py, the CT occurs even faster, which could be attributed to a more favorable configuration of the donor-acceptor pair, where TFP exhibits a closer energy match with the pyrene molecule. This is right energy alignment likely facilitates faster CT process.
To fully address this comment, we expanded the explanation in the revised version of the manuscript (See page 9), to make it clear to the readers.

Q3. Can the solution assembly be microscopically viewed? Or any strong other evidences be provided?

Response: We thank the reviewer for this suggestion. Upon the addition of a mixed solution of TCNB and Pyrene into ethanol/water, cocrystal formation becomes evident within seconds, indicating the successful assembly of monomers into cocrystals. To fully address this comment, we added photographs depicting various stages of the cocrystal formation process in the revised version of the SI (See Figure S1) and the corresponding descriptions in the revised version of the manuscript (See page 5).
